# Supplementary material for: Virus-Induced Flowering by Apple Latent Spherical Virus Vector: Effective Use to Accelerate Breeding of Grapevine
Source: Viruses. 2020 Jan 7;12(1):70. doi: 10.3390/v12010070 (PMC7019355; doi:10.3390/v12010070)
Supplement: Supplementary file 1 [file viruses-12-00070-s001.pdf]

Table S1. Primers used for construction and detection of ALSV vectors

Primer name	Accession no.	Sequence	Insert size (nt positions)
VvPDS-XhoI(+)	EU816356	5'-TAC ATC TCG AGA TTG AGC TGA ACA AAG ACG G-3'	201 (104-304)
VvPDS-BamHI(-)		5'-TAC ATG GAT CCC TCA AAC CAT ATA TGA ACA T-3'	
VvFT-XhoI(+)	EF157728	5'-TAC ATC TCG AGA TGC CTA GGG AAA GGG ATC C-3'	525 (41-565)
VvFT-SmaI(-)		5'-TAC ATC CCG GGT TAT GAT CTT CGA CCA CCC GAG C-3'	
VvTFL1A-SalI(+)	DQ871591	5'-TAC ATG TCG ACC CTC TTA TTG TTG GTA GAT-3'	201 (71-271)
VvTFL1A-MluI(-)		5'-TAC ATA CGC GTA TCA GGG TCT GTC ATA ATC AG-3'	
VvTFL1A- XhoI (+)	DQ871591	5'-TAC ATC TCG AGC CTC TTA TTG TTG GTA G-3'	201 (71-271)
VvTFL1A- SmaI (-)		5'-TAC ATC CCGGGATCAGGGTCTGTCATAATC-3'	
VvTFL1B-SalI(+)	DQ871592	5'-TAC ATG TCG ACC CCG AAG CCT AAC ATA GG-3'	183 (429-611)
VvTFL1B-MluI(-)		5'-TAC ATA CGC GTC TTG CAG CTG TTT CTC GT-3'	
VvTFL1B- XhoI (+)	DQ871592	5'-TAC ATC TCG AGA TGA CTG TTA CTT ACC AC-3'	201 (181-381)
VvTFL1B- SmaI (-)		5'-TAC ATC CGG GTG GAATGTCTGTCACTAT-3'	
VvTFL1C- XhoI (+)	DQ871593	5'-TACATCTCGAGCCAAGCCCTAGTGATCCATAC-3'	201 (223-413)
VvTFL1C- SmaI (-)		5'-TACATCCCGGGATGGTCTCTTGAAGCTGGTG-3'	
ALR2-999(+)		5'-GCT CTC TGT AGT TAT TCT GCA G-3'	for detection of ALSV
ALR2-1437(-)		5'-GAC CTT CTA GCA GAT TTG GG-3'	
ALR2-1418(+)		5'-CCC AAA TCT GCT AGA AGG TC-3'	
ALR2-1511(-)		5'-GCA AGG TGG TCG TGA-3'	
ALR1-6598(+)		5'-GTA CAT TCC TCC CAA TCA AAG-3'	
ALR1-6691(-)		5'-GGA TCA CGA GAA CAA ACT AG-3'	
VvPDS(+)	JQ319631	5'-CGT CCC AGT AAA CCA TTA GA-3'	for qRT-PCR
VvPDS(-)		5'-CCA CCT AAA ACA TCT CTT GC-3'	
VvEF1α(+)	XM_002279562.3	5'-GTC GGT TAC AAC CCT GAT AA-3'	
VvEF1α(-) f		5'-ATC ATG TCA AGA GCC TCA AG-3'	

Table S2. Contents of sugar, acid, and total anthocyanin in fruits of F1 hybrids, Ganebu, Crimson glory vine, and commercial cultivars of grapevine

Cultivar or F1 hybrid (line)	Contents of [1]		
	Sugar (Brix. %) ±SD	Acid (%) ±SD	Total anthocyanin (mg/g fw) ±SD
Ganebu	19.5 ±1.49	1.77 ±0.13	69.4 ±7.81
Ganebu x 'Nehelescol' (240-1)	19.2 ±0.67	1.88 ±0.34	45.9 ±1.73
'Cabernet Sauvignon' x Ganebu (4-23)	13.7 ±1 .63	1.73 ±0.22	59.2 ±0.45
Ganebu x 'Shine Muscat' (264-T31)	18.6 ±3,36	1.50 ±0.39	14.5 ±8.13
Yamabudo（*V. coignetiae*）	23.2±0.88	2.03±0.26	45.5±11.44
'Delaware'	22.3 ±0.72	0.57 ±0.12	7.7 ±1.59
'Merlot'	22.0 ±0.98	1.10 ±0.18	7.2 ±0.80
'Kyoho'	18.4 ±1.27	0.64 ±0.08	26.9 ±12.92
'Steuben'	20.5 ±0.68	0.39 ±0.06	36.0 ±3.99

[1] Total anthocyanin content in berry skins were shown as a malvidin 3-glucoside chloride equivalent mg/g fresh weight. The value was average from 5 - 10 berries.

Gapes of commercial cultivars were purchased in fruit shop and used analysis.
